# Statins Use in Patients with Cardiovascular Diseases and COVID-19 Outcomes: An Italian Population-Based Cohort Study

**DOI:** 10.3390/jcm11247492

**Published:** 2022-12-17

**Authors:** Ippazio Cosimo Antonazzo, Carla Fornari, Davide Rozza, Sara Conti, Raffaella Di Pasquale, Paolo Angelo Cortesi, Shaniko Kaleci, Pietro Ferrara, Alberto Zucchi, Giovanni Maifredi, Andrea Silenzi, Giancarlo Cesana, Lorenzo Giovanni Mantovani, Giampiero Mazzaglia

**Affiliations:** 1 Research Centre on Public Health, University of Milano-Bicocca, 20900 Monza, Italy; 2 IRCCS, Istituto Auxologico Italiano, 20145 Milan, Italy; 3 Health Protection Agency of Bergamo (ATS Bergamo), 24121 Bergamo, Italy; 4 Health Protection Agency of Brescia (ATS Brescia), 25124 Brescia, Italy; 5 General Directorate of Health Prevention, Ministry of Health, 00144 Rome, Italy

**Keywords:** COVID-19, statins, pharmacoepidemiology, public health, ICU access, mechanical ventilation, mortality

## Abstract

Background: The role of statins among patients with established cardiovascular diseases (CVDs) who are hospitalized with COVID-19 is still debated. This study aimed at assessing whether the prior use of statins was associated with a less severe COVID-19 prognosis. Methods: Subjects with CVDs infected with SARS-CoV-2 and hospitalized between 20 February 2020 and 31 December 2020 were selected. These were classified into two mutually exclusive groups: statins-users and non-users of lipid-lowering therapies (non-LLT users). The relationship between statins exposure and the risk of Mechanical Ventilation (MV), Intensive Care Unit (ICU) access and death were evaluated by using logistic and Cox regressions models. Results: Of 1127 selected patients, 571 were statins-users whereas 556 were non-LLT users. The previous use of statins was not associated with a variation in the risk of need of MV (Odds Ratio [OR]: 1.00; 95% Confidence Intervals [CI]: 0.38–2.67), ICU access (OR: 0.54; 95% CI: 0.22–1.32) and mortality at 14 days (Hazard Ratio [HR]: 0.42; 95% CI: 0.16–1.10). However, a decreased risk of mortality at 30 days (HR: 0.39; 95% CI: 0.18–0.85) was observed in statins-users compared with non-LLT users. Conclusions: These findings support the clinical advice for patients CVDs to continue their treatment with statins during SARS-CoV-2 infection.

## 1. Introduction

In March 2020 the World Health Organization (WHO) declared the Coronavirus disease 2019 (COVID-2019) outbreak a global pandemic [1,2]. Almost two years later, there have been more than 600 million cases and more than 6.5 million deaths worldwide [1].

Despite the exact mechanisms underlying severe COVID-19 remaining unknown, it has been suggested that during the response to SARS-CoV-2 the immune dysregulation and the high levels of pro-inflammatory cytokines might represent pivotal causes of tissue damage [3,4,5]. In this context, statins, which are usually used to treat dyslipidaemia, gained attention due to their potential role in COVID-19 prognosis [6,7,8]. Several studies highlighted their anti-inflammatory, immunomodulatory, antithrombotic, and antiviral properties that may translate into improved short- and long-term outcomes in COVID-19 patients [6,9,10,11].

Since the outbreak of COVID-19, several studies investigating the role of statins in COVID-19 patients consistently ruled out any beneficial effect [12,13,14,15,16,17,18,19,20,21,22,23]. However, contrasting evidence has been reported in patients with cardiovascular diseases (CVDs) who develop COVID-19. These subjects, on one hand, have been found to be associated with an increased risk of severe COVID-19 and worse clinical outcomes [24,25,26]; on the other hand, they may benefit from statins treatment because of their established protective role in CVDs prevention [27].

This study aimed to assess whether preadmission statin use in patients with CVDs who tested positive for SARS-CoV-2 and were hospitalized was associated with less severe COVID-19 prognosis, indicated by need for mechanical ventilation (MV), intensive care unit (ICU) access and death.

## 2. Materials and Methods

### 2.1. Study Design and Data Source

This is a cohort study based on data from the healthcare administrative database (HAD) of local Health Protection Agencies (HPA) of Bergamo (HPA-Bergamo) and Brescia (HPA-Brescia), Italy, that gather health data for people living in the relevant catchment areas (province of Bergamo and Brescia). The two HPAs cover a population of about 2.3 million inhabitants. The Italian HAD record all reimbursement data, linked at patient level by using an anonymized code. For this study we used the following database: (1) the SARS-CoV-2 swab registry; (2) the hospital discharge database (HDD) including information on admission and discharge dates and hospital diagnosis coded according to the ICD9-CM; (3) the drug dispensing registry, which includes information on dispensing date, doses and number of dispensed drugs by community or hospital pharmacies to subjects; (4) the chronic disease registry, which includes data on chronic disease; and (5) the inhabitants registry, which includes dates and reasons for entry and exit in the HPAs catchment area.

### 2.2. Study Population

The population under study included all inhabitants (≥18 years old) in the territory of Bergamo- and Brescia-HPA, Italy, with a CVD such as ischemic heart disease, peripheral vascular disease, cerebrovascular disease and heart failure, and who tested positive for SARS-CoV-2 and were hospitalized between 20 February 2020 and 31 December 2020. The date of first hospitalization was considered as the index date (ID). Individuals with less than one year of database history prior to the ID were excluded from the cohort. Finally, individuals were followed up until death or the end of the observational period (30 days after the ID), whichever came first.

### 2.3. Study Groups

Individuals included into the study were grouped into two mutually exclusive categories: statins users (those who were under statins treatment at ID or within 30 days prior to the ID), and lipid-lowering therapies (LLTs) non-users (those who were not treated with any lipid-lowering therapies within one year prior to the ID). Those who were under statins treatment within one year prior to the ID and 30 days prior to the ID were excluded from the cohort because they were considered to be past users. In addition, subjects exposed to other LTTs rather than statins were also excluded from the cohort. Therefore, patients exposed to statins (statins users) were compared with those not exposed to any LLTs.

### 2.4. Outcome

During follow-up, the occurrence of the following outcomes was investigated: need of MV, ICU admission as reported in the HDD and all-cause mortality at 14 and 30 days after ID, as reported in the HDD and/or health registry. For the need of MV and ICU access, only the occurrence of these outcomes was available; no information on the date of these events was recorded.

### 2.5. Patients’ Characteristics

For each selected individual, demographic characteristics such as sex and age were extracted at ID, whereas clinical information (comorbidities and drug exposure) were investigated in the period prior to the ID. Data on comorbidities were retrieved by using the chronic disease registry updated on 1 January 2020. In the chronic disease registry, each disease is assessed by merging data from different data sources such as pharmacy claims and inpatient and outpatient care. Therefore, for each selected individual the presence of the following comorbidities was evaluated: Alzheimer or dementia, respiratory diseases, hypertension, diabetes, chronic liver diseases, rheumatic diseases, cancer, and infection with human immunodeficiency virus (HIV). Data on concomitant drugs exposure within three months prior to the ID were assessed. In particular, the following exposures were investigated: anticoagulants, nonsteroidal anti-inflammatory drugs, chloroquine or hydroxychloroquine, corticosteroids for systemic use (plain) and immunosuppressant drugs. Finally, for those included in the statins users group, the type of statin exposure was extracted at ID. In particular, the following statins were investigated: atorvastatin, simvastatin, rosuvastatin, pravastatin, fluvastatin and lovastatin.

### 2.6. Statistical Analysis

The statistical estimates were shown as frequencies and percentages for categorical variables, mean and standard deviation (SD) for continuous variables and median (q_1_–q_3_) for counting variables. The differences between the two study groups were examined using the Pearson’s chi-square or Fisher test for categorical variables, and Student’s *t*-test or Wilcoxon test for continuous variables.

The association between statins use and mortality at 14 and 30 days after ID was assessed by using unadjusted and adjusted Cox proportional hazard regression. Results were expressed as Hazard Ratio (HR) with 95% confidence intervals (95% CI). The association between statins use and need of MV or ICU access was assessed using unadjusted and adjusted logistic regression models. Results were expressed as Odds Ratio (OR) and 95% CI. Both Cox and Logistic models were adjusted for demographic (i.e., sex and age) and clinical (i.e., comorbidities) characteristics.

To assess the potential impact of different statin exposure level on the study outcomes, we repeated the analysis by including the level of exposure to statins therapy in the statins group. Exposure was measured by the cumulative number of days during which the drug was available in the year prior to the ID divided by 365 days. Results were expressed as proportion of covered days (PDC); therefore, three categories were detected: low (PDC < 50%), intermediate (50–79%), and high (≥80%) [28]. Finally, to further investigate the robustness of the findings in respect to a possible residual confounding effect, subgroup analyses were carried out as follows: age (<65 years and ≥65 years), sex, presence of diabetes, which generally makes patients more prone to experience worse prognosis during COVID-19, and concomitant use of anticoagulants.

All statistical analyses were performed using R version 4.0.5 (the R Foundation for Statistical Computing, Vienna, Austria) and SAS version 9.4 (SAS Institute, Cary, NC, USA).

## 3. Results

### 3.1. Descriptive Statistics

**Figure 1:**
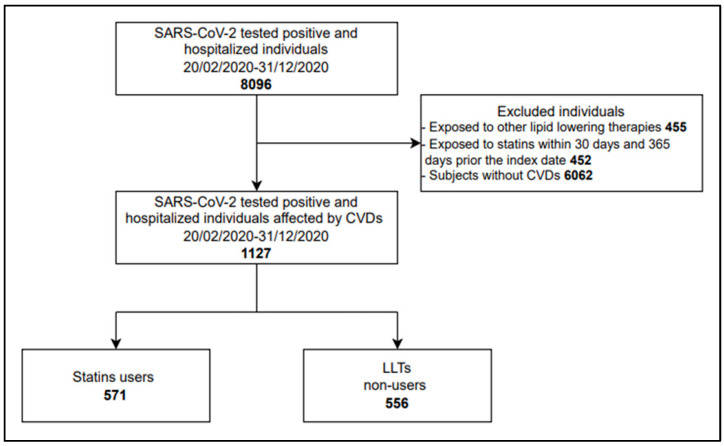
Study cohorts’ selection. LLTs: Lipid-lowering therapies.

Of the 8096 individuals who tested positive for SARS-CoV-2 and were hospitalized between 20 February 2020 and 31 December 2020, 1127 (13.9%) had at least one CVD and fulfilled inclusion criteria. Among them, 571 were identified as statin users and 556 were non-LLT users (Figure 1).

The demographic and clinical characteristics of the study population were reported in Table 1. Among the statins users, atorvastatin was the most prescribed (60.4%) medication, followed by simvastatin (20%), rosuvastatin (16.3%), pravastatin (2.1%), fluvastatinn (1.1%), and lovastatin (0.2%); 71% of users showed high adherence (PDC ≥ 80%) to the treatment in the year prior to the ID. Statin users were more likely to be male compared with LLT non-users (69% vs. 57%), whereas no age difference was observed between groups. Statins users also reported significantly higher prevalence of diabetes (40% vs. 26%) compared with non-LLT users (Table 1). They were also more likely to use anticoagulants in the three months prior to the ID (33% vs. 2%).

### 3.2. Outcomes

**Figure 2:**
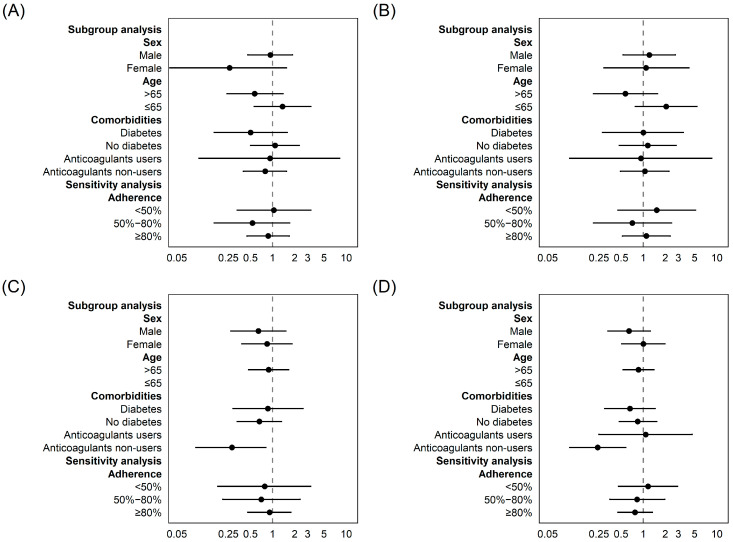
Subgroup and adherence analyses of ICU access risk (**A**), need of mechanical ventilation risk (**B**) and 14day (**C**) and 30-day (**D**) all-cause mortality in CVDs patients that tested positive for SARS-CoV-2 and were hospitalized. Legend: For anticoagulant-use group in panel (**C**) and ≤65 years group in (**C**,**D**), the estimation was not allowed due to absence of data.

Use of statins prior to hospitalization was not associated with significant changes in the risk of need of MV (OR: 1.00; 95% CI: 0.38–2.67) and ICU access (OR: 0.54; 95% CI: 0.22–1.32) (Table 2). Non-significant results for both outcomes were also confirmed in all the subgroup analyses (Figure 2). The use of statins was associated with not significant reduction of mortality at 14 days (HR: 0.42; 95% CI: 0.16–1.10) and with a statistically significant decreased risk of mortality at 30 days (HR: 0.39; 95% CI: 0.18–0.85) (Table 2). As reported in the stratified analyses (Figure 2), among non-users of anticoagulants we observed a significant decreased risk of mortality at both 14 days (HR: 0.28; 95% CI: 0.09–0.81) and at 30 days (HR: 0.24; 95% CI: 0.10–0.58) associated with statins use.

## 4. Discussion

In this retrospective cohort analysis of patients with CVDs who tested positive for SARS-CoV-2 and were hospitalized, statins use before hospital admission was not associated with a decreased risk of ICU access and MV need compared with non-LLT users. Similar results were found in different ages, genders, and levels of statins exposure, regardless of whether the participants had comorbidities. On the other hand, results suggested a protective role of statins on mortality at 30 days. Additionally, sensitivity analyses performed by selecting only patients with specific CVD (i.e., ischemic heart diseases, cerebrovascular diseases, heart failure) were consistent with the main results.

Our findings on ICU access and MV need are in line with those reported in most published studies, suggesting no association between statins use and variation in the risk of ICU access and/or need of MV in hospitalized COVID-19 patients with history of CVDs [29,30,31]. Indeed, the 61% decreased risk of mortality at 30 days in statin users is also consistent with the range of 30–70% lower mortality risk reported in previous studies [31,32,33] but it does contrast with other two studies reporting no association between statins use and in-hospital mortality [29,30].

CVDs, prominent risk factors for developing severe COVID-19, are commonly treated with statins. Therefore, in COVID-19 patients, a complex interplay between the effects of these diseases and statins can be hypothesized. For example, statins might inhibit either the main protease of SARS-CoV-2 with consequent alteration of its infectivity properties [34], or the expression of receptors (i.e., Toll-like receptors) on immune cells with consequent down-regulation of the activity of mediators (i.e., NF-κB) which are typically involved in inflammatory processes, cytokine storms and respiratory distress [8,35,36,37,38,39,40,41,42]. Furthermore, statins might cause a decrease of cholesterol levels in the plasma membranes, which causes alteration in the ACE2 assemblage, with consequent failure in SARS-CoV-2 internalization and egression from the cells [6,43,44]. Additionally, cholesterol is an important component for viral membrane formation and for the repair of the host’s membrane after the virus cycle is completed in order to prevent the loss of cell homeostasis. Therefore, a low level of cholesterol could be associated with a low ability of the virus to complete its vital cycle and to continue its replication in the host [44]. Finally, the antithrombotic effects of statins might reduce the risk of cardiovascular complications typically observed in COVID-19 patients, thus resulting in their improved survival [38].

In spite of these mechanistic hypotheses, the lack of effects on in-hospital outcomes (i.e., MV and ICU) suggests that statins have no direct effect on pathogenesis in COVID-19 patients across the different stages of disease [45]. Moreover, the improvement in mortality at 30 days, observed particularly among non-users of anticoagulants, might be consistent with the hypothesis that the major benefit of these medications accrues from treating the underlying disease in stable patients, such as those that do not need treatment with anticoagulants. This is also in line with the well-known benefit of statins in improving long-term outcomes among patients with elevated risk for CVDs [46].

The main strength of this study is the completeness of data included in the used database. In fact, the Italian healthcare system, funded by taxes, provides equal access to healthcare service to all residents in the country regardless of their socioeconomic status. Furthermore, in Italy, statins are reimbursed by the national healthcare system and can be dispensed by pharmacies to general population only with a medical prescription. The findings of this study should also be interpreted in light of some limitations. Although the Italian HAD collects data on reimbursed services, it does not include data on in-hospital patient management or clinical data such as severity of COVID-19 disease, blood sample parameter results and other laboratory tests (i.e., microbiological tests). Second, the Italian pharmacy claim registry does not include data on over-the-counter (OTC) medication; therefore, we were not able to adjust the analysis by including this type of data. Finally, although we adjusted the analysis by including several measurable parameters such as comorbidities and previous drug exposure, as already observed for other similar studies conducted using the same database [47], we cannot completely rule out the presence of residual unmeasurable confounding (i.e., clinical and lifestyle parameters) that can impact the observed findings. In fact, it is possible to speculate that individuals under preventive treatment (i.e., statins treatments) are more prone to have healthy behavior in terms of diet, physical activity, alcohol and tobacco consumption. Therefore, if these characteristics are not properly considered in the analysis, we may observe a potential overestimation of the beneficial effects of statin treatment [48].

## 5. Conclusions

In this Italian cohort study, exposure to statins in patients with CVDs hospitalized forSARS-CoV-2 infection did not affect the risk of ICU access or need of MV. On the contrary, previous use of statins was associated with a lower risk of mortality at 30 days in these patients. These findings support the clinical advice for patients with cardiovascular and/or metabolic diseases to continue their treatment with statins during SARS-CoV-2 infection.

## Figures and Tables

**Table 1:** Demographic and clinical characteristics of patients with CVDs who tested positive for SARS-CoV-2 and were hospitalized between 20 February 2020 and 31 December 2020.

Characteristics	Statins User (N = 571)	Non-LLT User (N = 556)	Total (N = 1127)
N	571	556	1127
Female	176 (30.8%)	239 (43.0%) *	415 (36.8%)
Age mean ± SD	76.8 (8.5)	76.3 (12.3)	76.6 (10.5)
Age groups			
0–44	0 (0.0%)	7 (1.3%) *	7 (0.6%)
45–64	59 (10.3%)	89 (16%) *	148 (13.1%)
≥65	512 (89.7%)	460 (82.7%) *	972 (86.2%)
Previous statins exposure, N (%)			
Atorvastatin	345 (60.4%)	---	---
Simvastatin	114 (20.0%)	---	---
Rosuvastatin	93 (16.3%)	---	---
Pravastatin	12 (2.1%)	---	---
Fluvastatin	6 (1.1%)	---	---
Lovastatin	1 (0.2%)	---	---
Statins adherence groups, N (%)			
≥80%	406 (71.1%)	---	---
50–79%	105 (18.4%)	---	---
<50%	60 (10.5%)	---	---
Comorbidity, N (%)			
Alzheimer/Dementia	12 (2.1%)	41 (7.4%) *	53 (4.7%)
Respiratory disease	71 (12.4%)	83 (14.9%)	154 (13.7%)
Other cardiac diseases	268 (46.9%)	269 (48.4%)	537 (47.6%)
Hypertension	470 (82.3%)	398 (71.6%) *	868 (77%)
Dyslipidemia	477 (83.5%)	63 (11.3%) *	540 (47.9%)
Diabetes	228 (39.9%)	146 (26.3%) *	374 (33.2%)
Chronic liver disease	66 (11.6%)	85 (15.3%)	151 (13.4%)
Rheumatic disease	19 (3.3%)	9 (1.6%)	28 (2.5%)
Cancer	66 (11.6%)	80 (14.4%)	146 (13.0%)
HIV	1 (0.2%)	1 (0.2%)	2 (0.2%)
Concomitant therapies within 3 months, N (%)			
Anticoagulants	190 (33.3%)	12 (2.2%) *	202 (17.9%)
Corticosteroids for systemic use	63 (11.0%)	65 (11.7%)	128 (11.4%)
Nonsteroidal anti-inflammatory drugs	40 (7.0%)	37 (6.7%)	77 (6.8%)
Chloroquine, hydroxychloroquine	13 (2.3%)	12 (2.2%)	25 (2.2%)
Immunosuppressant drugs	4 (0.7%)	9 (1.6%)	13 (1.2%)
N. different ATC (5 level), median (q_1_–q_3_)	5 (4–7)	3 (1–5) *	4 (2–6)

* *p*-value ≤ 0.05; LLTs: Lipid-lowering therapies.

**Table 2:** Use of statins and ICU access, mechanical ventilation (MV), and mortality in patients with CVDs who tested positive for SARS-CoV-2 and were hospitalized.

Outcome	Number of Events	Unadjusted Relative Risk *	Adjusted Relative Risk *^,§^
ICU access			
Non-LLT user	30 (5.4%)	Reference	Reference
Statins user	31 (5.4%)	1.01 (0.60, 1.69)	0.54 (0.22, 1.32)
Mechanical ventilation			
Non-LLT user	21 (3.8%)	Reference	Reference
Statins user	29 (5.1%)	1.36 (0.77, 2.42)	1.00 (0.38, 2.67)
Mortality at 14 days			
Non-LLT user	32 (5.8%)	Reference	Reference
Statins user	19 (3.3%)	0.57 (0.32, 1.01)	0.42 (0.16, 1.10)
Mortality at 30 days			
Non-LLT user	51 (9.2%)	Reference	Reference
Statins user	31 (9.2%)	0.58 (0.37, 0.91)	0.39 (0.18, 0.85)

LLTs: Lipid-lowering therapies. Bold: statistically significant Relative Risk. * It is Hazard Ratio for hospitalization and mortality and Odds Ratio for ICU access and mechanical ventilation. ^§^ The estimate is adjusted by: age, sex, comorbidities, concomitant use of anticoagulants, NSAIDs, chloroquine/hydroxycloroquine and immunosuppressants.

## Data Availability

Not applicable.
